# Supplementary material for: Partially Redundant Actin Genes in Chlamydomonas Control Transition Zone Organization and Flagellum-Directed Traffic
Source: Cell Rep. Author manuscript; Available in PMC 2019 May 29. (PMC6541019; doi:10.1016/j.celrep.2019.04.087)
Supplement: 1 [file NIHMS1530018-supplement-1.pdf]

Cell Reports, Volume *27*

Supplemental Information

Partially Redundant Actin Genes in *Chlamydomonas* Control Transition Zone Organization and Flagellum-Directed Traffic

Brittany Jack, David M. Mueller, Ann C. Fee, Ashley L. Tetlow, and Prachee Avasthi

Supplemental Material

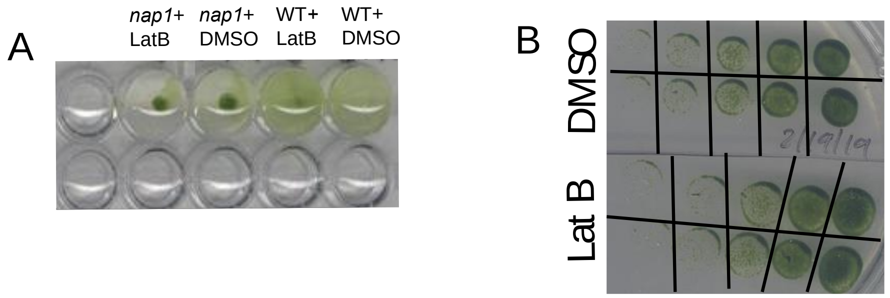

C

Cell/Volume Quantification		
Treatment	wild-type	*nap1*
1% DMSO	3.04 x 106 cell/mL	3.48 x 10^6 cell/mL
10 μM LatB	31.8 x 106 cell/mL	2.50 x 10^6 cell/mL

D

% Flagellation		
Treatment	wild-type	*nap1*
1% DMSO	72 %	73 %
10 μM LatB	87 %	82 %

Supplemental Figure 1. *Chlamydomonas* strains survive LatB treatment for at least up to 5 hours Related to Figure 2. A. wild-type and *nap1* strains treated with 10µM Lat B for 5 hours. LatB was washed out and an aliquot of the cells were added to 99 µL of liquid TAP media immediately following experiment. 5 days after re-suspension in TAP media growth was assessed and we see growth of all strains indicating wild-type and *nap1* can survive LatB treatment for at least 5 hours. **B**. wild-type and *nap1* strains treated with 10µM Lat B for 5 hours. LatB was washed out and an aliquot of the cells were plated on 1.5% TAP plates. 5 days after plating on TAP plates growth was assessed and we see growth of all strains indicating wild-type and nap1 can survive LatB treatment for at least 5 hours. **C.** Cell/volume quantification 5 days after LatB washout to show survival. **D**. Percent flagellation quantification 5 days after LatB washout cells.

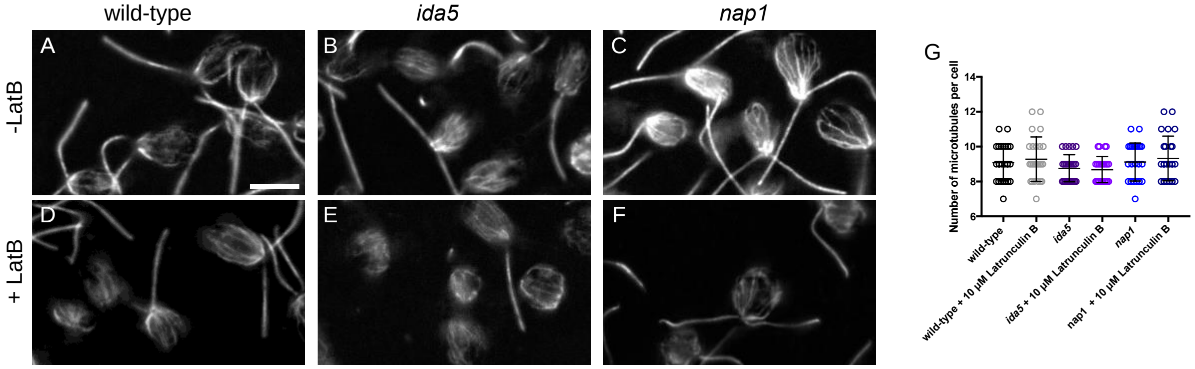

Supplemental Figure 2. Actin disruption does not cause alteration in microtubule number Related to Figure 2. A. Wild-type cells without LatB treatment. **B**. *ida5* mutants without LatB treatment. **C**. *nap1* mutants cells without LatB treatment. **D**. Wild-type cells with LatB treatment show no difference in number of microtubules. **E**. *ida5* mutants cells with LatB treatment show no difference in microtubule number.

F. *nap1* mutant cells with LatB treatment show no difference in LatB microtubule number. **G**. Quantification of number of microtubules per cell.
