# Supplementary material for: Partially Redundant Actin Genes in Chlamydomonas Control Transition Zone Organization and Flagellum-Directed Traffic
Source: Cell Rep. Author manuscript; Available in PMC 2019 May 29. (PMC6541019; doi:10.1016/j.celrep.2019.04.087)
Supplement: 2 [file NIHMS1530018-supplement-2.pdf]

Cell Reports

Article

Partially Redundant Actin Genes in *Chlamydomonas* Control Transition Zone Organization and Flagellum-Directed Traffic

Graphical Abstract

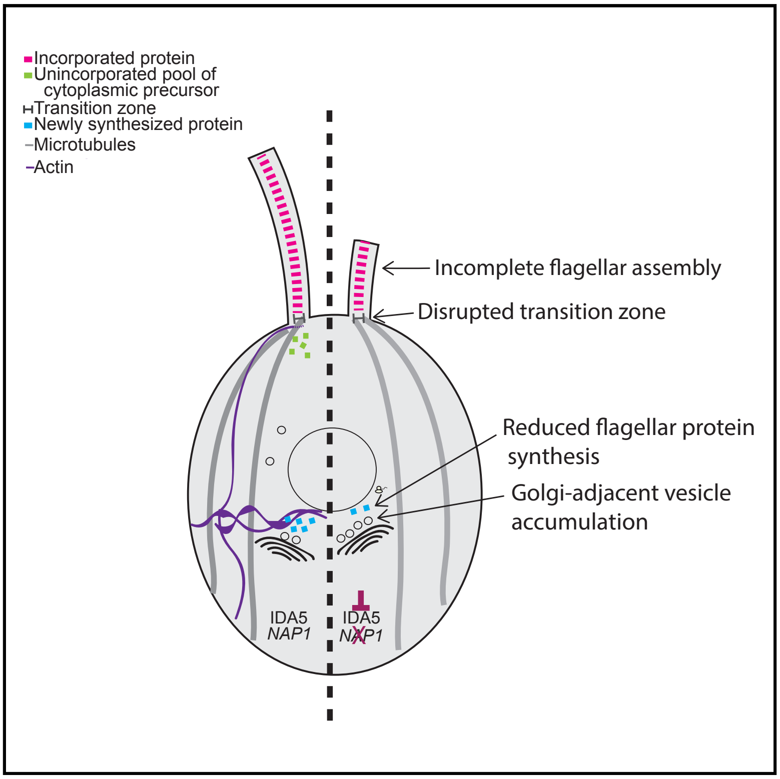

Highlights

- At least one filamentous actin required for full flagellar assembly
- Transition zone flagellar gate affected upon actin disruption
- Actin required for normal flagellar protein synthesis
- Golgi-adjacent vesicles accumulate upon total actin loss

Authors

Brittany Jack, David M. Mueller, Ann C. Fee, Ashley L. Tetlow, Prachee Avasthi

Correspondence

pavasthi@kumc.edu

In Brief

Prevailing knowledge tells us that intracellular trafficking of flagellar proteins occurs on microtubule tracks. In this study, Jack et al. discover that filamentous actin is required for full flagellar assembly, normal flagellar protein synthesis, an intact flagellar gating region, and vesicle transport in *Chlamydomonas reinhardtii*.

Jack et al., 2019, Cell Reports *27*, 2459–2467
May 21, 2019 © 2019 The Author(s).
https://doi.org/10.1016/j.celrep.2019.04.087

CellPress

OPEN ACCESS
CellPress

Partially Redundant Actin Genes in *Chlamydomonas* Control Transition Zone Organization and Flagellum-Directed Traffic

Brittany Jack,[1] David M. Mueller,[1] Ann C. Fee,[2] Ashley L. Tetlow,[1] and Prachee Avasthi[1,3,4,*]
[1]Department of Anatomy and Cell Biology, University of Kansas Medical Center, Kansas City, KS 66160, USA
[2]University of Missouri-Kansas City, School of Medicine, Kansas City, MO 64110, USA
[3]Department of Ophthalmology, University of Kansas Medical Center, Kansas City, KS 66160, USA
[4]Lead Contact
*Correspondence: pavasthi@kumc.edu
https://doi.org/10.1016/j.celrep.2019.04.087

SUMMARY

The unicellular green alga *Chlamydomonas reinhardtii* is a biflagellated cell with two actin genes: one encoding a conventional actin (IDA5) and the other encoding a divergent novel actin-like protein (NAP1). Here, we probe how actin redundancy contributes to flagellar assembly. Disrupting a single actin allows complete flagellar assembly. However, when disrupting both actins using latrunculin B (LatB) treatment on the *nap1* mutant background, we find that actins are necessary for flagellar growth from newly synthesized limiting flagellar proteins. Under total actin disruption, transmission electron microscopy identified an accumulation of Golgi-adjacent vesicles. We also find that there is a mislocalization of a key transition zone gating and ciliopathy protein, NPHP-4. Our experiments demonstrate that each stage of flagellar biogenesis requires redundant actin function to varying degrees, with an absolute requirement for these actins in transport of Golgi-adjacent vesicles and flagellar incorporation of newly synthesized proteins.

INTRODUCTION

Assembly and composition of the eukaryotic flagellum (also known as the cilium) are critical for signaling and development in most cell types in the human body. The flagella of the green alga *Chlamydomonas reinhardtii* are essentially identical to the cilia of mammalian cells and provide an excellent model to study cell signaling, cell motility, and regulation of ciliary assembly. To date, the known mechanisms dictating the behavior of these organelles are dependent largely on the microtubule cytoskeleton. The flagellum is composed of microtubules that extend from a microtubule organizing center known as the basal body, and flagellar assembly requires control of microtubule dynamics. Trafficking from sites of cellular protein synthesis to their ultimate destination in flagella is also thought to occur on microtubule tracks (Tai et al., 1999).

However, evidence for the role of actin, another major cytoskeletal component, in ciliary regulation is increasing. In mammalian cells, disruption of actin leads to increases in both ciliary length and percentage of ciliated cells (Kohli et al., 2017; Sharma et al., 2011), which may be due to roles for actin in basal body migration, docking, and stabilization (Kim et al., 2010; Pan et al., 2007; Park et al., 2008; Tu et al., 2017; Yeyati et al., 2017). A recent study showed loss of myosin-Va, an actin-based motor protein involved in trafficking of secretory vesicles from the Golgi to the plasma membrane, resulted in reduced ciliation. Disruption of myosin-Va function stops the formation of the elongated ciliary membrane. This new result suggests that actin and myosin-Va are required for microtubule-dependent trafficking of preciliary and ciliary vesicles to the base of the cilia and therefore necessary for ciliogenesis (Wu, et. al, 2018). Actin is also required for vesicle budding in the endocytic pathway (Girao et al., 2008), which may influence ciliary assembly, as there is a trafficking pathway connecting the endocytic compartments to a vesicular compartment involved in ciliary assembly (Kim et al., 2010). Membrane remodeling for both ciliary exocytosis (Nager et al., 2017) and ciliary resorption are actin-dependent processes (Saito et al., 2017). In summary, the current understanding is that actin networks (which potentially block cortical access of basal bodies and ciliary proteins) inhibit cilium formation and elongation in mammalian cells but are also required for membrane trafficking to support ciliogenesis. Here, using an algal model system, we show a broader requirement for filamentous actin in flagellar protein synthesis, trafficking, and incorporation of proteins into an assembling flagellum.

Using *Chlamydomonas reinhardtii* as a model to interrogate flagellar dynamics, we are able to induce flagellar severing on demand to allow synchronous and successive rounds of flagellar regeneration. *Chlamydomonas* has two actin genes: one that encodes a conventional actin (IDA5) and another that encodes a novel actin-like protein (NAP1) with ~65% homology to mammalian actin (Hirono et al., 2003). *Chlamydomonas* cells treated with cytochalasin D, an actin polymerization inhibitor, exhibit flagellar shortening and regrowth upon washout of the drug, suggesting a role for actin in flagellar maintenance (Dentler and Adams, 1992). *ida5* mutants and myosin-inhibited cells had impaired flagellar motor recruitment to basal bodies, impaired entry of motors into the flagellar compartment, and ultimately reduced initial

This is an open access article under the CC BY license (http://creativecommons.org/licenses/by/4.0/).

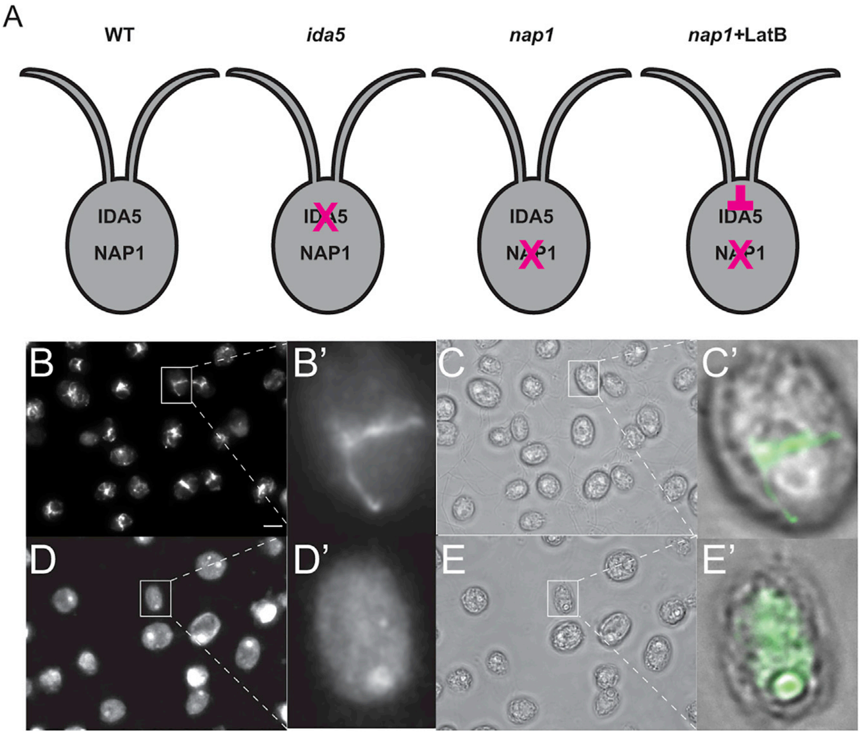

Figure 1. Latrunculin B Disrupts Actin Filaments on *nap1* Mutant Background

(A) Genetic and chemical approach to investigate the role of actin in flagellar assembly.

(B) *nap1* mutant cells stained with phalloidin show filamentous actin. Scale bar represents 5 μm.

(B′) Zoom (4×) of cell outlined in white box in (B).

(C) Brightfield image of *nap1* mutant cells prior to LatB treatment.

(C′) Zoom (4×) of cell outlined in white box in (C) with overlay of phalloidin staining.

(D) *nap1* mutant cells treated with 10 μM LatB and stained with phalloidin do not show filamentous actin.

(D′) Zoom (4×) of cell outlined in white box in (D).

(E) Brightfield image of *nap1* mutant cells after 2 h of LatB treatment.

(E′) Zoom (4×) of cell outlined in white box in (E) with overlay of phalloidin staining.

flagellar growth rate (Avasthi et al., 2014). Despite defects in flagellar protein recruitment and flagellar assembly in *ida5* conventional actin mutants, these mutants ultimately grow flagella to wild-type lengths. Because *ida5* mutant flagella eventually reach wild-type length, the degree to which actin is required is still in question.

In this study, we asked if more severe flagellar biogenesis phenotypes were masked by redundant roles of the second *Chlamydomonas* actin NAP1, as *NAP1* expression increases upon IDA5 loss or disruption (Onishi et al., 2016). Using genetic and chemical perturbations of both *Chlamydomonas* actins, we found that the two actin genes have overlapping functions that include flagellar protein synthesis and composition of the flagellar gate. We also found an absolute requirement for these actins in flagellar incorporation of newly synthesized flagellar proteins. Here, we explore the role of IDA5 and NAP1 as well as their importance for trafficking flagellar proteins and building flagella.

RESULTS

Simultaneous Disruption of All Filamentous Actins within *Chlamydomonas* Cells

To investigate if *NAP1* contributes to the actin-dependent flagellar assembly functions previously identified for the conventional actin *IDA5* (Avasthi et al., 2014), we obtained a *nap1*-null mutant strain, which was isolated on the basis of its sensitivity to latrunculin B (LatB), a conventional actin depolymerizing agent (Onishi et al., 2016). Here we use both a genetic and chemical approach to understand the role of actin in flagellar assembly (Figure 1A). LatB has been shown to disrupt filamentous actin in *Chlamydomonas reinhardtii* (Avasthi et al., 2014; Onishi et al., 2016). Treatment with LatB for 10 min is sufficient to disrupt actin filaments in wild-type cells (Onishi et al., 2016). To confirm that LatB disrupts actin filaments under our experimental conditions, we fixed and stained the *nap1* mutant strain with phalloidin, a filamentous actin probe (Figure 1). Using our optimized actin visualization protocol (Craig et al., 2019), we see actin filaments are present in the *nap1* mutants prior to drug treatment (Figure 1B). Upon LatB treatment, these actin filaments disappear (Figure 1D).

Chlamydomonas cells are known to upregulate NAP1 upon LatB treatment, resulting in the return of filamentous signal, which represents the LatB-insensitive NAP1 population (Onishi et al., 2016, 2018). However, in *nap1* mutants, this upregulation cannot occur. Therefore, we do not see filaments return 2 h post-LatB treatment (Figure 1D). These results indicate that during the experiments presented here, we are testing phenotypes under conditions of no filamentous actin (neither IDA5 nor NAP1) in LatB-treated *nap1* mutant cells.

Flagellar Length Maintenance and Assembly Requires at Least One Functional Actin

Cells with genetic disruption of *NAP1* and chemical perturbation of *IDA5* filaments lack all filamentous actins and ultimately cannot survive for long periods of time (Onishi et al., 2016). However, acute perturbation using LatB on the *nap1* mutant background allows us to probe the functions of both actins on the shorter timescales needed for assessing flagellar dynamics (Figure S1). When wild-type and *nap1* mutants are treated with 10 μM LatB, wild-type flagella shorten but eventually recover, whereas *nap1* mutant flagella continue to shorten (Figure 2A). To test if flagellar defects are due to indirect effects of actin disruption on microtubule organization, we labeled microtubules and confirmed that there were no gross defects in microtubule organization or microtubule number in LatB-treated *nap1* mutants (Figure S2). These data suggest

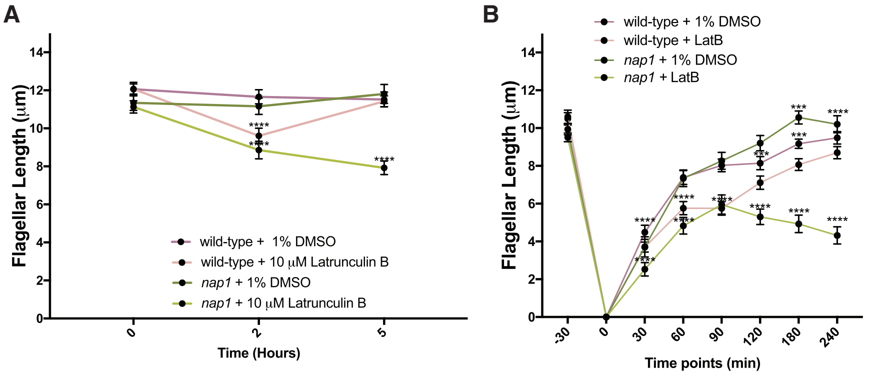

Figure 2. Actin Filaments Are Necessary for Flagella Length Maintenance and Full Flagellar Assembly

(A) Cells of each type were exposed to 10 μM LatB. Wild-type (WT) flagella shorten but recover, while nap1 mutants + LatB cannot recover. Error bars represent 95% confidence intervals.

(B). When all actin is disrupted in nap1 mutant cells, the flagella grow only to half length. Error bars represent 95% confidence interval.

***$p < 0.001$ and ****$p < 0.0001$; N = 1, n = 50.

that some form of filamentous actin, either *IDA5* or *NAP1*, is required for flagellar maintenance.

ida5 mutants initially assemble their flagella more slowly but ultimately reach wild-type length (Avasthi et al., 2014). However, given that loss of all actins prevented flagellar maintenance (Figure 2A), we investigated if flagella could fully assemble when both actins are perturbed by deflagellating wild-type and *nap1* mutants via pH shock and regenerating flagella in the presence of LatB. When *IDA5* is disrupted on the *nap1* mutant background, flagella cannot grow beyond half-length of a typical flagellum (Figure 2B).

Flagellar Protein Synthesis Is Reduced upon Actin Disruption

One explanation for the inability of flagella to grown beyond half-length growth in LatB-treated *nap1* mutants is that the cell is not capable of synthesizing new protein under actin-depleted conditions. Cells treated with the protein synthesis inhibitor cycloheximide following severing were previously shown to grow only to half length (Rosenbaum et. al, 1969). This half-length growth in cycloheximide uses already synthesized flagellar proteins and demonstrates that synthesis of new limiting flagellar protein is required for full flagellar assembly.

Because flagellar regeneration in *Chlamydomonas* requires new flagellar protein synthesis to support assembly to full length, and because LatB-treated *nap1* mutant flagella only assemble to half length (similar to growth in cycloheximide when no proteins can be synthesized), we next tested if actin loss blocked flagellar protein synthesis. Actin has known roles in transcription (Miralles and Visa, 2006) and may be involved in the deflagellation-induced expression of flagellar proteins. As the amount of available protein prior to deflagellation is enough to assemble a half-length flagellum, synthesis of new flagellar proteins can be quantified using cumulative flagellar growth beyond half length using an assay diagramed in Figure 3A and first described by Lefebvre et al. (1978). To test actin's effect on the amount of new protein synthesis at different time points following flagellar severing, *nap1* mutants are deflagellated and treated with 10 μM LatB to allow protein synthesis and flagellar assembly to proceed. Cells are deflagellated a second time at 30 minute intervals, LatB is washed out, and all of the already synthesized limiting flagellar proteins are incorporated into flagella upon addition of cycloheximide. By comparing the cumulative length of flagella under actin-perturbed conditions with half-length flagellar growth in cycloheximide (when zero protein synthesis takes place), we can infer the extent to which new limiting flagellar proteins were synthesized in the LatB treatment period (Jack and Avasthi, 2018). We see that loss of all actins does indeed reduce the amount of limiting flagellar proteins synthesized (Figure 3B) using this method in which flagellar length is a proxy for limiting flagellar protein synthesis.

Actin Disruption Blocks All Flagellar Growth When Flagellar Precursor Proteins Are Depleted

If limiting flagellar proteins can be synthesized under actin-depleted conditions, the question remains as to why these flagella cannot assemble beyond half length. We asked if the flagellar growth represents incorporation of only the existing pool of cytoplasmic flagellar proteins but not newly synthesized flagellar proteins. To test this, we depleted the limiting flagellar proteins in the cytoplasmic pool prior to flagellar assembly under actin-disrupting conditions. A schematic of this experiment is shown in Figure 4A. In this precursor protein-depleted condition, the cell must synthesize proteins *de novo* and traffic and incorporate them into the flagella. In this context, complete disruption of actin blocks all flagellar growth (Figure 4B). This suggests that (1) the flagella can reach half length in LatB-treated *nap1* mutants exclusively from incorporation of the existing pool of flagellar proteins, and (2) some form of actin is an absolute requirement for newly synthesized proteins to be incorporated into growing flagella.

Golgi-Adjacent Vesicles Accumulate upon Actin Disruption

To assemble a flagellum, all the necessary components must arrive at the base of the cilia, as there are no ribosomes within the ciliary compartment (Diener et al., 2015). We have shown that, without actin, there is no flagellar growth under conditions in which all components needed to build a flagellum are depleted and must be newly synthesized, trafficked, and incorporated (Figure 4). The question remains as to why this newly synthesized protein (Figure 3) cannot be incorporated into a flagellum. To examine if there are gross abnormalities in cell or Golgi morphology that could be the cause of this trafficking and

OPEN
ACCESS
CellPress

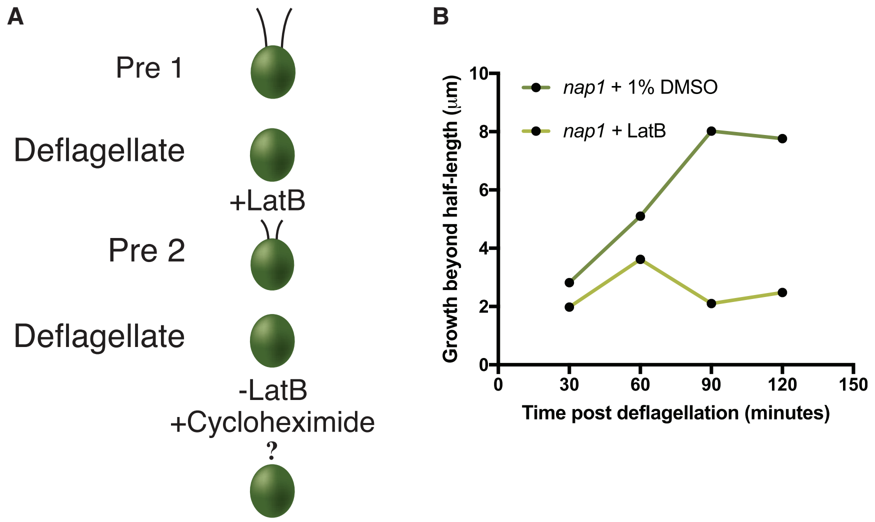

Figure 3. Flagellar Protein Synthesis Is Reduced When Actins Are Disrupted

(A) Schematic representation of new protein synthesis assay.

(B) Cells were deflagellated in the presence of 10 μM LatB and deflagellated a second time washing out the LatB and adding 10 μg/mL cycloheximide. This process of double deflagellation allows the quantification of newly synthesized limiting flagellar protein when actins are disrupted. Fewer new flagellar proteins are synthesized upon disruption of both actins. For all measurements, N = 1, n = 30.

incorporation problem, *nap1* mutant flagella were severed to induce conditions that require additional protein synthesis and trafficking. These cells were visualized using transmission electron microscopy (TEM), and we found no noticeable abnormalities in Golgi cisternae in control cells (Figures 5A and 5B, magenta arrows). However, under actin-deficient conditions, we saw a dramatic accumulation of Golgi-adjacent vesicles (Figures 5C and 5D, magenta arrows). To determine the extent of the accumulation and identify any additional defects, we quantified the number (Figure 5E) and size (Figure 5F) of these vesicles. The 18 analyzed control and actin-deficient cells had 109 and 225 total vesicles, respectively, while the difference in diameter was not significant.

Actin Disruption Produces Flagellar Gating Defects

During regeneration in LatB-treated *nap1* mutants (Figure 2B), we noticed that the flagellar growth to half length appeared slower than cells containing both actins. To test whether this represents a defect in incorporating proteins from the existing pool of flagellar precursors, we simultaneously treated *nap1* mutants with cycloheximide and LatB following deflagellation. LatB-treated cells (both wild-type cells that take time to upregulate *NAP1* and *nap1* mutants) incorporate the existing pool of flagellar precursors at a slower rate than cells with intact actins (Figure 6R). We also previously found that during flagellar assembly, there was a small range of flagellar lengths (7–9 μm) within which flagellar motor recruitment to basal bodies in *ida5* mutants was comparable with controls but motor entry into flagella was reduced (Avasthi et al., 2014). These data, in conjunction with slow incorporation of existing precursors, suggest a role for actin in gating of material already accumulated at the flagellar base. A region thought to be critical for gating functions is the transition zone at the base of flagella, which has connectors between the microtubule core and flagellar membrane. The transition zone also houses proteins found to regulate the composition of flagella (Awata et al., 2014). Actin itself is found in the transition zone of flagella (Diener et al., 2015) and may function to transport or anchor the transition zone gating proteins. *NPHP-4*, a gene mutated in the cilium-related kidney disease nephronophthisis, is a crucial component of the ciliary gate that controls entry of both membrane-associated and soluble proteins (Awata et al., 2014). We used a strain expressing HA-tagged NPHP-4 to test the effects of functional actin loss on NPHP-4 localization. In control cells stained with anti-HA antibody, NPHP-4 localizes at two apical spots at the base of flagella (Figure 6A). In cells treated with LatB for 10 and 30 min, we saw a dramatic loss of apical NPHP-4 localization (Figures 6E and 6I). Occasionally we could see some remaining apical staining, and the results are quantified in Figure 6Q. These results demonstrate that acute disruption of actin with LatB treatment (at a time point prior to *NAP1* upregulation) causes a reorganization of the transition zone for loss of a known transition zone gating protein, NPHP-4. Extended treatment of LatB (2 h), a condition in which NAP1 is upregulated, shows a recovery of NPHP-4 to the apical part of the cell (Figures 6M and 6Q, gray bars). For orientation purposes, acetylated tubulin and bright-field images were used to show NPHP-4 localization in the cell. This suggests that NAP1 can perform this function of conventional actin at the transition zone.

DISCUSSION

In this study, we acutely disrupt all filamentous actin in *Chlamydomonas* through treatment of *nap1* mutants with LatB (Figure 1). Recent transcriptomic analyses show that depolymerization of F-actin by LatB treatment in these cells induces an upregulation not only of NAP1 but several hundred other genes, including a general upregulation of the ubiquitin proteasome system to monitor actin filament integrity and prevent dominant-negative effects on NAP1 by monomeric IDA5 (Onishi et al., 2016, 2018). The upregulation of these genes may influence the phenotypes seen in this study and should be considered as potential contributing factors. However, *ida5* mutants do not show significant upregulation of the same genes (Onishi et al., 2018) suggesting that this is not a broad non-specific response to LatB treatment but rather a response to actin filament depolymerization. Furthermore, *ida5* mutants in the absence of LatB treatment also showed impairment in flagellar protein recruitment to the base of flagella (Avasthi et al., 2014), which is consistent with our model suggesting roles for actins in post-Golgi flagellum-directed transport (Figure 7).

OPEN ACCESS
CellPress

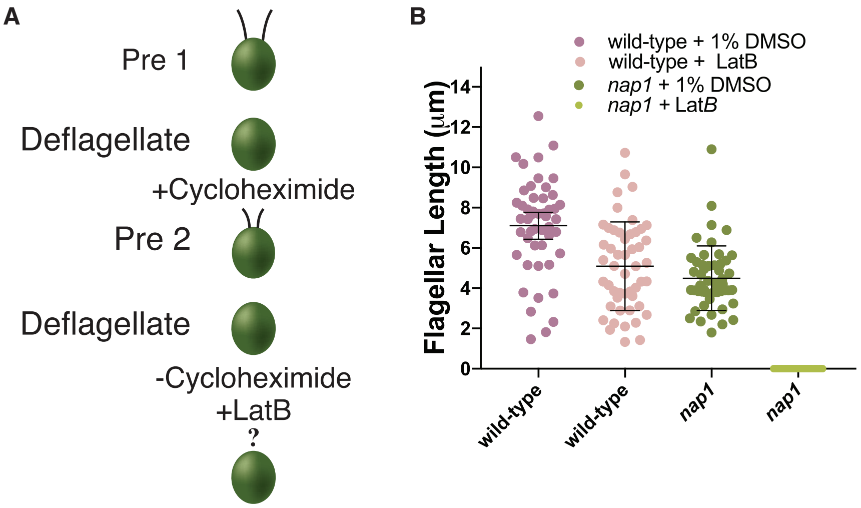

Figure 4. Functional Actin Is Necessary for Incorporation of Newly Synthesized Flagellar Protein

(A) Schematic of new flagellar protein incorporation assay.

(B) Cells were deflagellated in the presence of 10 μg/mL of cycloheximide and deflagellated a second time washing out the cycloheximide and adding 10 μM LatB. This process of double deflagellation allowed for the incorporartion of the exisiting available proteins and the assessment of newly synthesized, trafficked, and incorporated flagellar protein. There is no flagellar growth using newly synthesized protein in *nap1* mutants when IDA5 actin is disrupted. Error bars are 95% confidence interval (N = 1, n = 50).

Our data support a requirement of at least one form of *Chlamydomonas* actin for flagellar length maintenance and full flagellar assembly. Anterograde flagellar motor complexes, which are required for assembly of the flagellum, use newly recruited proteins from the cell body pool (Wingfield et al., 2017). With constant turnover and a demand for continuous recruitment to the base of flagella, it appears that actin plays a role in this recruitment (Avasthi et al., 2014). We find functional actin is required for normal flagellar protein synthesis and normal incorporation of existing proteins (Figure 7). Importantly, we found that actin-deficient cells cannot grow flagella at all when new flagellar proteins must be (1) synthesized, (2) trafficked, and (3)

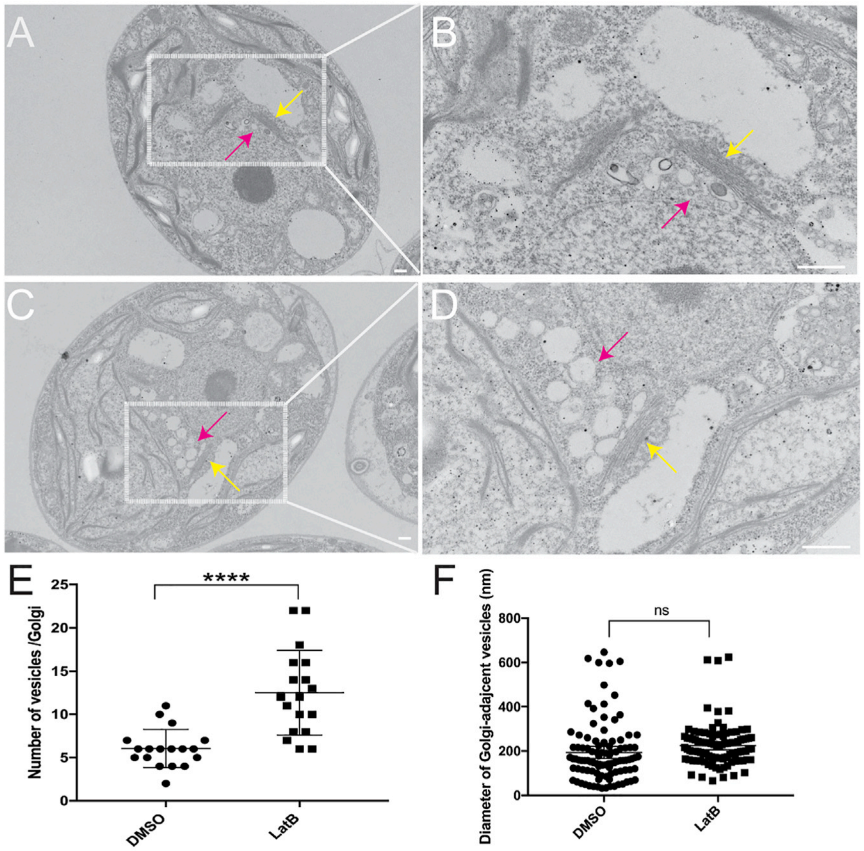

Figure 5. Actin Depletion Results in Accumulation of Golgi-Adjacent Vesicles upon Flagellar Regeneration

(A) *nap1* cells treated with DMSO 30 min post-deflagellation at 4,000× magnification. Scale bar represents 200 nm.

(B) *nap1* cells treated with DMSO 30 min post-deflagellation at 10,000× magnification. This is an independent image of the area outlined in (A) taken at a higher magnification. Scale bar represents 500 nm.

(C) *nap1* cells treated with LatB 30 min post-deflagellation at 4,000× magnification.

(D) *nap1* cells treated with LatB 30 min post-deflagellation. Independent image of area outlined in (C) taken at 10,000× magnification. Yellow arrows point to the Golgi apparatus, and magenta arrows point to Golgi-adjacent vesicles.

(E) Quantification of number of vesicles per Golgi (N = 2, n = 18).

(F) Quantification of diameter of Golgi-adjacent vesicles (N = 2, n = 100).

Error bars represent 95% confidence interval. ****p = 0.0019.

OPEN ACCESS
CellPress

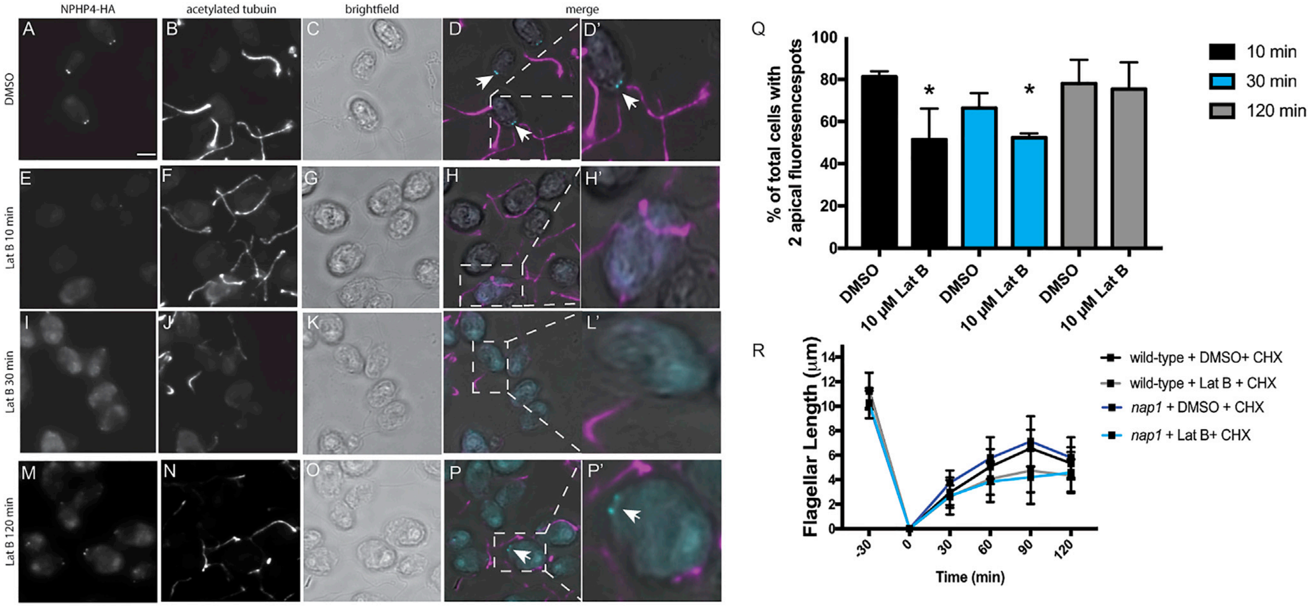

Figure 6. The Transition Zone Gating Protein NPHP-4 and Incorporation of Flagellar Proteins Are Affected upon Disruption of Chlamydomonas Actins

(A) Cells expressing HA-tagged NPHP-4 were stained with HA primary antibody, and localization is seen at the base of the flagella.
(B) Cells expressing HA-tagged NPHP-4 were stained with acetylated tubulin primary antibody to indicate flagella.
(C) Brightfield image of cells expressing HA-tagged NPHP-4.
(D) Merge image of HA, acetylated tubulin, and brightfield.
(D′) Zoom (2×) inset of cell in (D).
(E) Cells treated with 10 μM LatB for 10 min were stained with the HA antibody. Mislocalization of the NPHP-4 protein is seen throughout the cell.
(F) Cells expressing HA-tagged NPHP-4 were stained with acetylated tubulin primary antibody, and localization is seen in the flagella.
(G) Brightfield image of cells expressing HA-tagged NPHP-4.
(H) Merge image of HA, acetylated tubulin, and brightfield.
(H′) Zoom (2×) inset of cell in (H).
(I) Cells treated with 10 μM LatB for 30 min were stained with the HA antibody. Mislocalization of the NPHP-4 protien is seen throughout the cell.
(J) Cells expressing HA-tagged NPHP-4 were stained with acetylated tubulin primary antibody to indicate flagella.
(K) Brightfield image of cells expressing HA-tagged NPHP-4.
(L) Merge image of HA, acetylated tubulin, and brightfield.
(L′) Zoom (2×) inset of cell in (L).
(M) Cells treated with 10 μM LatB for 120 min were stained with the HA antibody.
(N) Cells expressing HA-tagged NPHP-4 were stained with acetylated tubulin primary antibody, and localization is seen in the flagella.
(O) Brightfield image of cells expressing HA-tagged NPHP-4.
(P) Merge image of HA, acetylated tubulin, and brightfield.
(P′) Zoom (2×) inset of cell in (P) (recovery of apical NPHP-4 localization).
(Q) Percentage of cells with two apical fluorescence spots upon NPHP-4 labeling is quantified (*$p < 0.05$, N = 3).
(R) Cycloheximide is used to stop new protein synthesis. The addition of LatB (or 1% DMSO control) is to test the effect actin disruption has on incorporating flagellar protein. Flagellar lengths were assessed every 30 min for 2 h. Error bars represent 95% confidence. Scale bar represents 5 μm. N = 3, n = 100.

incorporated (Figure 4). However, although actins are involved, some level of flagellar protein synthesis and incorporation occurs (given the reduced but non-zero values for LatB-treated *nap1* mutants in Figures 3B and 4). Together, these data support a model in which actin is an absolute requirement for the release of newly synthesized protein into the cytoplasmic protein pool that can be used for flagellar growth. Flagellar membrane proteins and vesicles originate from the Golgi (Nachury et al., 2010; Rohatgi and Snell, 2010). Furthermore, in *Chlamydomonas*, collapse of the Golgi upon treatment with brefeldin A results in a shortening of the flagella, suggesting that limiting protein or membrane destined for the flagella are being exported from the Golgi (Dentler, 2013). Therefore, we reasoned that in the absence of actin, limiting components cannot be properly recruited, sorted, or released from the Golgi. Our TEM results show that Golgi morphology is not dramatically altered, but there is an accumulation of what appear to be secretory vesicles adjacent to the *trans* face of the Golgi (the *trans* face identified by narrower cisternae [Engel et al., 2015] in close proximity to larger secretory vesicles). Given the *trans* Golgi vesicle accumulation and previous studies implicating a role of myosin in the trafficking of flagellum-directed cargo (Avasthi et al., 2014), we propose a model in which actomyosin-dependent transport is required for either short- or long-range trafficking of flagellum-bound vesicles after protein synthesis. Further studies are required to evaluate the role of actin in flagellar protein sorting and

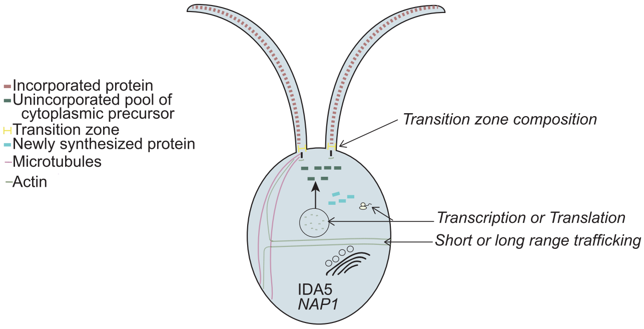

Figure 7. Model of Actin-Dependent Ciliary Functions

Loss of all functional actins via depolymerization of IDA5 actin in nap1 mutants results in no flagellar growth using newly synthesized protein. Disruption of both actins causes slower protein incorporation and less newly synthesized flagellar protein but cannot account for the inability to grow flagella. We hypothesize that functional actin is required for short- or long-range trafficking of newly synthesized protein to the flagellar base.

trafficking, including the role of individual myosins that have been implicated by us (Avasthi et al., 2014) and others (Assis et al., 2017).

We show here that actin is involved in flagellar protein synthesis (Figure 3). Actins are known to localize to the nucleus (Pederson and Aebi, 2002) and interact with transcription factors, chromatin remodeling complexes, and RNA polymerases (Miralles and Visa, 2006). However, it is not well defined whether all or a subset of transcripts require actin function. Given the extensive interaction of actin with core transcriptional machinery, perhaps it is more unexpected that some flagellar protein synthesis can indeed occur in the absence of functional actins. The identity of the proteins that are reduced under these conditions is still unknown. It remains to be determined via proteomics and transcriptomics whether our assay reflects a decrease in translation or transcription.

Last, we found a significant disruption of NPHP-4 transition zone localization (Figures 5B and 5D). In *Chlamydomonas*, actin was identified through biochemical purification as a transition zone protein and may serve as a scaffold in the region. Although NPHP-4 turnover appears more static in *Chlamydomonas* cells (Awata et al., 2014), actin and myosins found in the peri-basal body region (Assis et al., 2017; Avasthi et al., 2014) may be involved in turnover of transition zone components in mammalian cells in which the NPHP-4 turnover rate is high (Takao et al., 2017). *Nphp-4* mutants also show decreased membrane protein entry into flagella, suggesting that the protein is important in regulating flagellar composition. Membrane-associated proteins in *C. elegans* (Williams et al., 2011) and soluble housekeeping proteins in *Chlamydomonas* (Awata et al., 2014) also inappropriately accumulate within cilia in *nphp-4* mutants, suggesting that there is a more general dysregulation of ciliary gating upon NPHP-4 loss. Finally, *nphp-4* mutants in *C. elegans* have ultrastructural abnormalities (Jauregui et al., 2008; Lambacher et al., 2016). Therefore, at minimum, actin disruption, which significantly affects NPHP-4 localization, is expected to encompass the full range of ciliary phenotypes associated with NPHP-4 loss.

Chlamydomonas Nphp-4 mutants exhibit slow flagellar assembly that ultimately reaches wild-type flagellar lengths (Awata et al., 2014), similar to *ida5* mutants (Avasthi et al., 2014). Is NPHP-4 loss then the primary cause of slow initial flagellar assembly in *ida5* mutants? We found that NPHP-4 reappears at the basal bodies after extended treatment with LatB on *nap1* mutant cells. These data suggest that NAP1 is able to compensate for the functions of IDA5 at the transition zone. Although NAP1 and IDA5 have some redundant functions, we still see slower rates of assembly in the *ida5* mutant, in which NAP1 is expressed and further upregulated during regeneration. NAP1 expression levels may be insufficient early on in regeneration to support proper NPHP-4 localization. Alternatively, other factors outside of transition zone disruption may be responsible for early regeneration defects in actin-disrupted cells.

Our previous work (Avasthi et al., 2014) and the data presented here allow us to isolate specific steps of flagellar assembly that require normal actin dynamics. Actin has many cellular functions, including in organelle morphology, transcription, membrane dynamics, and polarized trafficking. We are now beginning to see how these conserved actin functions can dramatically influence flagellar biogenesis, a process previously thought to be controlled primarily through microtubule regulation.

STAR★METHODS

Detailed methods are provided in the online version of this paper and include the following:

- KEY RESOURCES TABLE
- CONTACT FOR REAGENT AND RESOURCE SHARING
- EXPERIMENTAL MODEL AND SUBJECT DETAILS
- METHOD DETAILS
  - Inhibitor Treatment and Flagellar Length Measurements
  - Phalloidin Staining
  - Flagellar Regeneration
  - Immunofluorescence Microscopy of NPHP4-HAC
  - New protein synthesis quantification
  - Electron Microscopy
  - Golgi-adjacent vesicle and size quantification
  - Cell Survival in LatB
  - Microtubule Staining
  - Microtubule Quantification
- QUANTIFICATION AND STATISTICAL ANALYSIS

SUPPLEMENTAL INFORMATION

Supplemental Information can be found online at https://doi.org/10.1016/j.celrep.2019.04.087.

ACKNOWLEDGMENTS

We would like to thank Masayuki Onishi, John Pringle, and Fred Cross for providing the *nap1* mutant. Thank you to members of the Avasthi lab for troubleshooting and manuscript feedback. Thank you to Evan Craig, whose optimized phalloidin staining protocol was used in this study. Thanks to Bill Dentler for helpful comments and critical reading of the manuscript. This work was

funded by grants P20 GM104936-09 and 1R35GM128702-01 (to P.A.) and NSF GRFP grant 1518767 (to B.J.).

AUTHOR CONTRIBUTIONS

Conceptualization, B.J., D.M.M., A.C.F., A.L.T., and P.A.; Methodology, B.J., D.M.M., A.C.F., A.L.T., and P.A.; Investigation, B.J., D.M.M., A.C.F., and A.L.T.; Writing – Original Draft, B.J. and P.A.; Writing – Review & Editing, B.J., D.M.M., A.C.F., A.L.T., and P.A.; Visualization, B.J., D.M.M., A.C.F., A.L.T., and P.A.; Supervision, P.A.; Project Administration, P.A.; Funding Acquisition, B.J. and P.A.

DECLARATION OF INTERESTS

The authors declare no competing interests.

Received: October 4, 2018
Revised: December 3, 2018
Accepted: April 18, 2019
Published: May 21, 2019

REFERENCES

Assis, L.H., Silva-Junior, R.M., Dolce, L.G., Alborghetti, M.R., Honorato, R.V., Nascimento, A.F., Melo-Hanchuk, T.D., Trindade, D.M., Tonoli, C.C., Santos, C.T., et al. (2017). The molecular motor Myosin Va interacts with the cilia-centrosomal protein RPGRIP1L. Sci. Rep. *7*, 43692.

Avasthi, P., Onishi, M., Karpiak, J., Yamamoto, R., Mackinder, L., Jonikas, M.C., Sale, W.S., Shoichet, B., Pringle, J.R., and Marshall, W.F. (2014). Actin is required for IFT regulation in *Chlamydomonas* reinhardtii. Curr. Biol. *24*, 2025–2032.

Awata, J., Takada, S., Standley, C., Lechtreck, K.F., Bellvé, K.D., Pazour, G.J., Fogarty, K.E., and Witman, G.B. (2014). NPHP4 controls ciliary trafficking of membrane proteins and large soluble proteins at the transition zone. J. Cell Sci. *127*, 4714–4727.

Craig, E.W., Mueller, D.M., Schaffer, M., Engel, B.D., and Avasthi, P. (2019). The elusive actin cytoskeleton of a green alga expressing both conventional and divergent actins. bioRxiv. https://doi.org/10.1101/554279.

Dentler, W. (2013). A role for the membrane in regulating *Chlamydomonas* flagellar length. PLoS ONE *8*, e53366.

Dentler, W.L., and Adams, C. (1992). Flagellar microtubule dynamics in *Chlamydomonas*: cytochalasin D induces periods of microtubule shortening and elongation; and colchicine induces disassembly of the distal, but not proximal, half of the flagellum. J. Cell Biol. *117*, 1289–1298.

Diener, D.R., Lupetti, P., and Rosenbaum, J.L. (2015). Proteomic analysis of isolated ciliary transition zones reveals the presence of ESCRT proteins. Curr. Biol. *25*, 379–384.

Engel, B.D., Schaffer, M., Albert, S., Asano, S., Plitzko, J.M., and Baumeister, W. (2015). In situ structural analysis of Golgi intracisternal protein arrays. Proc. Natl. Acad. Sci. U S A *112*, 11264–11269.

Girao, H., Geli, M.I., and Idrissi, F.Z. (2008). Actin in the endocytic pathway: from yeast to mammals. FEBS Lett. *582*, 2112–2119.

Hirono, M., Uryu, S., Ohara, A., Kato-Minoura, T., and Kamiya, R. (2003). Expression of conventional and unconventional actins in Chlamydomonas reinhardtii upon deflagellation and sexual adhesion. Eukaryot. Cell *2*, 486–493.

Jack, B., and Avasthi, P. (2018). Erratum to: Chemical screening for flagella-associated phenotypes in Chlamydomonas reinhardtii. Methods Mol. Biol. *1795*, E1.

Jauregui, A.R., Nguyen, K.C., Hall, D.H., and Barr, M.M. (2008). The Caenorhabditis elegans nephrocystins act as global modifiers of cilium structure. J. Cell Biol. *180*, 973–988.

Kim, J., Lee, J.E., Heynen-Genel, S., Suyama, E., Ono, K., Lee, K., Ideker, T., Aza-Blanc, P., and Gleeson, J.G. (2010). Functional genomic screen for modulators of ciliogenesis and cilium length. Nature *464*, 1048–1051.

Kohli, P., Höhne, M., Jüngst, C., Bertsch, S., Ebert, L.K., Schauss, A.C., Benzing, T., Rinschen, M.M., and Schermer, B. (2017). The ciliary membrane-associated proteome reveals actin-binding proteins as key components of cilia. EMBO Rep. *18*, 1521–1535.

Lambacher, N.J., Bruel, A.L., van Dam, T.J., Szymańska, K., Slaats, G.G., Kuhns, S., McManus, G.J., Kennedy, J.E., Gaff, K., Wu, K.M., et al. (2016). TMEM107 recruits ciliopathy proteins to subdomains of the ciliary transition zone and causes Joubert syndrome. Nat. Cell Biol. *18*, 122–131.

Lefebvre, P.A., Nordstrom, S.A., Moulder, J.E., and Rosenbaum, J.L. (1978). Flagellar elongation and shortening in Chlamydomonas. IV. Effects of flagellar detachment, regeneration, and resorption on the induction of flagellar protein synthesis. J. Cell Biol. *78*, 8–27.

Miralles, F., and Visa, N. (2006). Actin in transcription and transcription regulation. Curr. Opin. Cell Biol. *18*, 261–266.

Nachury, M.V., Seeley, E.S., and Jin, H. (2010). Trafficking to the ciliary membrane: how to get across the periciliary diffusion barrier? Annu. Rev. Cell Dev. Biol. *26*, 59–87.

Nager, A.R., Goldstein, J.S., Herranz-Perez, V., Portran, D., Ye, F., Garcia-Verdugo, J.M., and Nachury, M.V. (2017). An actin network dispatches ciliary GPCRs into extracellular vesicles to modulate signaling. Cell *168*, 252–263.14.

Onishi, M., Pringle, J.R., and Cross, F.R. (2016). Evidence that an unconventional actin can provide essential F-actin function and that a surveillance system monitors F-actin integrity in Chlamydomonas. Genetics *202*, 977–996.

Onishi, M., Pecani, K., Jones, T., 4th, Pringle, J.R., and Cross, F.R. (2018). F-actin homeostasis through transcriptional regulation and proteasome-mediated proteolysis. Proc. Natl. Acad. Sci. U S A *115*, E6487–E6496.

Pan, J., You, Y., Huang, T., and Brody, S.L. (2007). RhoA-mediated apical actin enrichment is required for ciliogenesis and promoted by Foxj1. J. Cell Sci. *120*, 1868–1876.

Park, T.J., Mitchell, B.J., Abitua, P.B., Kintner, C., and Wallingford, J.B. (2008). Dishevelled controls apical docking and planar polarization of basal bodies in ciliated epithelial cells. Nat. Genet. *40*, 871–879.

Pederson, T., and Aebi, U. (2002). Actin in the nucleus: what form and what for? J. Struct. Biol. *140*, 3–9.

Rohatgi, R., and Snell, W.J. (2010). The ciliary membrane. Curr. Opin. Cell Biol. *22*, 541–546.

Rosenbaum, J.L., Moulder, J.E., and Ringo, D.L. (1969). Flagellar elongation and shortening in Chlamydomonas. The use of cycloheximide and colchicine to study the synthesis and assembly of flagellar proteins. J. Cell Biol. *41*, 600–619.

Saito, M., Otsu, W., Hsu, K.S., Chuang, J.Z., Yanagisawa, T., Shieh, V., Kaitsuka, T., Wei, F.Y., Tomizawa, K., and Sung, C.H. (2017). Tctex-1 controls ciliary resorption by regulating branched actin polymerization and endocytosis. EMBO Rep. *18*, 1460–1472.

Schindelin, J., Arganda-Carreras, I., Frise, E., Kaynig, V., Longair, M., Pietzsch, T., Preibisch, S., Rueden, C., Saalfeld, S., Schmid, B., et al. (2012). Fiji: an open-source platform for biological-image analysis. Nat. Methods *9*, 676–682.

Sharma, N., Kosan, Z.A., Stallworth, J.E., Berbari, N.F., and Yoder, B.K. (2011). Soluble levels of cytosolic tubulin regulate ciliary length control. Mol. Biol. Cell *22*, 806–816.

Tai, A.W., Chuang, J.Z., Bode, C., Wolfrum, U., and Sung, C.H. (1999). Rhodopsin's carboxy-terminal cytoplasmic tail acts as a membrane receptor for cytoplasmic dynein by binding to the dynein light chain Tctex-1. Cell *97*, 877–887.

Takao, D., Wang, L., Boss, A., and Verhey, K.J. (2017). Protein interaction analysis provides a map of the spatial and temporal organization of the ciliary gating zone. Curr. Biol. *27*, 2296–2306.e3.

Tu, F., Sedzinski, J., Ma, Y., Marcotte, E.M., and Wallingford, J.B. (2017). Protein localization screening in vivo reveals novel regulators of multiciliated cell development and function. J. Cell Sci. *131*, jcs206565.

Williams, C.L., Li, C., Kida, K., Inglis, P.N., Mohan, S., Semenec, L., Bialas, N.J., Stupay, R.M., Chen, N., Blacque, O.E., et al. (2011). MKS and NPHP modules cooperate to establish basal body/transition zone membrane associations and ciliary gate function during ciliogenesis. J. Cell Biol. *192*, 1023–1041.

Wingfield, J.L., Mengoni, I., Bomberger, H., Jiang, Y.Y., Walsh, J.D., Brown, J.M., Picariello, T., Cochran, D.A., Zhu, B., Pan, J., et al. (2017). IFT trains in different stages of assembly queue at the ciliary base for consecutive release into the cilium. eLife *6*, e26609.

Wu, C.T., Chen, H.Y., and Tang, T.K. (2018). Myosin-Va is required for preciliary vesicle transportation to the mother centriole during ciliogenesis. Nat. Cell Biol. *20*, 175–185.

Yeyati, P.L., Schiller, R., Mali, G., Kasioulis, I., Kawamura, A., Adams, I.R., Playfoot, C., Gilbert, N., van Heyningen, V., Wills, J., et al. (2017). KDM3A coordinates actin dynamics with intraflagellar transport to regulate cilia stability. J. Cell Biol. *216*, 999–1013.

STAR★METHODS

KEY RESOURCES TABLE

REAGENT or RESOURCE	SOURCE	IDENTIFIER
Antibodies		
Atto-Phalloidin 488	Sigma Aldrich	49409-10Nmol
Rat- anti-HA	Sigma Aldrich	11867423001; RRID:AB_390918
Acetylated-alpha tubulin	Cell Signaling	5335T; RRID:AB_10544694
Alexa Fluor 594 AffiniPure F(ab’) Fragment Goat Anti-Rabbit	Jackson Laboratories	111-586-003; RRID:AB_2338066
Alexa Fluor 594 AffiniPure F(ab’) Fragment Goat Anti-Rat	Jackson Laboratories	112-545-003; RRID:AB_2338351
Chemicals, Peptides, and Recombinant Proteins		
Latrunculin B	Sigma Aldrich	L5288-1MG
DMSO	Sigma Aldrich	D2650-5x5mL
Cycloheximide	Sigma Aldrich	C1988-1G
HEPES	Corning	61-034-RM
25% Glutaraldehyde	EMS	16220
16% paraformaldehyde	EMS	15710
NH_4Cl	Sigma Aldrich	254134-5G
$MgSO_4 \cdot 7H_2O$	Fisher Scientific	M63-500
$CaCl_2$	Fisher Scientific	10035-048
K_2PO_4	Fisher Scientific	P285-3
KH_2PO_4	Fisher Scientific	P288-500
Acetic acid	Fisher Scientific	A38-212
Hutner’s Trace Elements	Chlamydomonas Resource Center	C123
Granulated agar	Difco	214530
Experimental Models: Organisms/Strains		
CC 125 mt +	Chlamydomonas Center	N/A
CC-5115 mt -	Chlamydomonas Center	N/A
nap1 mutant	Fred Cross, Masa Onishi, and John Pringle	N/A
Software and Algorithms		
Graph Pad Prism 7	Graph Pad	N/A
Adobe Illustrator	Adobe	N/A
Image J	Schindelin et al., 2012	http://imagej.net
FIJI	Schindelin et al., 2012	http://fiji.sc

CONTACT FOR REAGENT AND RESOURCE SHARING

Further information and requests for resources and reagents should be directed to and will be fulfilled by the Lead Contact, Prachee Avasthi (pavasthi@kumc.edu).

EXPERIMENTAL MODEL AND SUBJECT DETAILS

The *Chlamydomonas* strains were obtained from the *Chlamydomonas* stock center (CC 125 mt+ and CC-5115 mt-) and the *nap1* mutant (mt-) was a generous gift from Fred Cross, Masayuki Onishi, and John Pringle. *Chlamydomonas* strains (CC 125, CC-3420, CC-5115, and the nap1 mutant) were all grown on 1.5% Tris-Acetate Phosphate Agar plates under constant blue (450-475 nm) and red light (625-660 nm) using the LumiBar (Lumi Grow Inc.) for 3-5 days. Strains were maintained on 1.5% Tris-Acetate Phosphate agar plates using the LumiBar. Cells were grown in liquid TAP media for 18 hours overnight using the LumiBar prior to each experiment.

METHOD DETAILS

Inhibitor Treatment and Flagellar Length Measurements

All strains were grown in liquid TAP medium for 18 hours prior to incubation with 10 μM Latrunculin B or 10 μg/mL Cycloheximide for indicated times. Cells were fixed in 1% glutaraldehyde and imaged by DIC microscopy at 40 X magnification. Flagellar lengths were measured using ImageJ software by line segment tracing and spline fitting.

Phalloidin Staining

Cells were grown in liquid TAP media 18 hours prior to the experiment. Cells were incubated with either 10 μM LatB or 1% DMSO for 2 hours. Cells were fixed to coverslips with 4% paraformaldehyde and permeabilized with acetone. Cells were stained with atto-phalloidin for 15 minutes (optimized time for bright signal) and washed with PBS prior to mounting (Craig et al., 2019).

Flagellar Regeneration

Flagellar regeneration was induced by deflagellating cells via pH shock by adding 60μL of 0.5N acetic acid followed by 70 μL of 0.5N KOH to 1 mL of cells in liquid TAP. Cells were fixed with 1% glutaraldehyde at 0, 30, 60, 90, 120, and 240 minutes. Cells were imaged by DIC microscopy at 40 X magnification. Flagellar lengths were measured using ImageJ software by line segment tracing and spline fitting. Double deflagellation experiments for new protein incorporation were performed as described above (Figure 2A). The cells were deflagellated via pH shock and treated with 10μg/mL of Cycloheximide for 2 hours. The cells were deflagellated a second time via pH shock and the cycloheximide was washed out and 10μM LatB was added. The cells were treated for 4 hours and samples were taken every 30 minutes and fixed with 1% glutaraldehyde. Flagella lengths were measured using ImageJ software by line segment tracing and spline fitting.

Immunofluorescence Microscopy of NPHP4-HAC

Cells were grown in liquid TAP media 18 hours prior to the experiment. Cells were incubated with either 10 μM LatB or 1% DMSO for 10, 30, and 120 min. Cells were fixed to coverslips with methanol fixation and washed once with 1X PBS. 100% block was added to the coverslips for 30 min and then replaced with block with 10% normal goat serum for an additional 30 mins. The primary antibodies, rat anti-HA antibody (Sigma) at 1:1000 dilution and rabbit anti-acetylated alpha tubulin at 1:1000 dilution antibody (Cell Signaling Technology) were added to the coverslips and placed in the 4°C overnight. The primary antibody was removed and 4 X 5 min wash in 1 X PBS. The anti-rat and anti-rabbit secondary antibodies (Sigma) were added to the coverslips at 1:1000 dilution for 1 hour. The secondary antibodies were removed and another 4 X 5 min wash in 1 X PBS. Coverslips were mounted with Fluoromount-G. Slides were visualized on Nikon TiS microscope on the FITC, TRITC, and brightfield channels.

New protein synthesis quantification

Two consecutive deflagellations were performed to quantify the amount of newly synthesized flagellar protein. Cells were deflagellated via pH shock in the presence of 10 μM LatB to allow protein synthesis under actin depleted conditions. The first deflagellation runs for a total of 2 hours but cells are deflagellated a second time every 30 minutes where the LatB is washed out and 10μg/mL cycloheximide is added in to prevent any new protein synthesis. The addition of cycloheximide allows only protein that was synthesized during LatB treatment to be incorporated into the assembling flagellum. Growth beyond half- length is quantified as newly synthesized protein. Quantification of newly synthesized protein was quantified using the below equation (Jack and Avasthi, 2018). Samples were taken every 30 min post deflagellation 1 at 30, 60, 90, and 120 min and fixed in 1% glutaraldehyde and 2 hours following the second deflagellation and fixed in 1% glutaraldehyde. Flagella lengths were measured using ImageJ software by line segment tracing and spline fitting.

$$new\ \ protein\ \ synthesis = \left(Y_{length\ after\ 1st\ deflag} + X_{final\ time\ pt.\ length\ 2nd\ deflag.}\right) - Z_{length\ after\ regen\ in\ CHX}$$

Electron Microscopy

For thin sections, cells were deflagellated via pH shock by adding 60μL of 0.5N acetic acid followed by 70 μL of 0.5N KOH to 1 mL of cells in liquid TAP. Cells regenerated flagella for 30 min and were then fixed in an equal volume of 2% glutaraldehyde for 15-20 min at room temp. The cells were gently pelleted using 1X G for 10 min and the supernatant was removed. Cells were gently re-suspended in 1% GLUT, 20mM sodium cacodylate and then fixed for 1 hr at room temp and overnight at 4 degrees. The staining protocol was followed according to Dentler and Adams (1992).

Golgi-adjacent vesicle and size quantification

To quantify the number of Golgi-adjacent vesicles in the TEM micrographs the Golgi was identified and all surrounding vesicles were counted using the cell counter plug-in on Fiji. To determine any size difference in the vesicles the diameter was measured using the segmented line tool in Fiji and the pixel measurement was changed to nm.

Cell Survival in LatB

Wild-type and *nap1* mutants were grown in liquid culture 18 hours and underwent the double deflagellation experiment for new protein incorporation. Following the experiment 1 μL of cells were added to 99 μL of liquid TAP media in a 96 well plate. An image was taken immediately after the experiment and 5 days following to assess for growth. Growth of all strains indicates these cells can survive LatB treatment for at least 5 hours. Cell/volume quantification was done using a hemocytometer and cell counter plug-in on Fiji. Images were taken of cells on a DIC microscope for % flagellation quantification and flagella were counted using cell counter plug-in on Fiji.

Microtubule Staining

Cells were grown in TAP medium 24 hours prior to treatment. The cells were treated with 10 μM Lat B for 30 minutes. The cells were then extracted into Eppendorf tubes and centrifuged for 2 minutes at 7500 *rcf.* The supernatant was removed and replaced with an equal amount of MT buffer. MT Buffer: 30 mM HEPES (pH 7.2), 3mM EGTA, 1mM MgSO4, and 25 mM KCl in ddH20. Cells were adhered to a poly-Lysine-treated coverslip for five minutes. The coverslips were then fixed 4%paraformaldehyde, diluted in MT buffer, for five minutes. Next, the coverslips were treated with 200μL of 0.5% NP40, also diluted in MT buffer. The cells underwent methanol extraction at −20°C for 5 minutes. The coverslips were placed in a humidified chamber and treated with 100μL of 100% block (5% BSA and 1% cold water fish gelatin in 1X PBS) for 30 minutes at room temperature. The solution was tilted off and 100μL of 10% Normal Goat Serum (diluted in 100% block) were applied, the coverslips sat for 30 minutes at room temperature, in the humidified chamber. The cells received a solution of primary antibody (monoclonal anti-mouse alpha tubulin1:100 purchased from Sigma) diluted in 20% block. The cells received primary antibody treatment in the humidified chamber overnight at −4°C. The following day, the cells were washed three times, for ten minutes each time, in 1X PBS, then treated for thirty minutes with secondary antibody (1:1000 goat-anti-mouse-Alexa 488) at room temperature in the humidified chamber. Again, the coverslips cells were washed three times, for ten minutes each time, in 1X PBS. Finally, the coverslips were mounted on glass slides with 7μL of Fluoromount-G and imaged in the FITC channel, taking 0.2μM step Z stack images on a Nikon TiS microscope.

Microtubule Quantification

After staining cells with phalloidin using the described method, FITC images were acquired using a Nikon Eclipse Ti microscope. The images were saved as multipage tifs (slices were 0.2 microns apart with around 30 slices per image). Quantification was done on images prior to deconvolution to ensure counting of faint microtubules but we are showing deconvolved images in the figure for clarity. Images were then individually opened in the ImageJ software where the first step was to convert the stack to hyperstack and to change the color of the image. Despite being taken under the FITC filter, the images became black and white upon transfer to the software. The color was changed back to green as it was more pronounced against the black background of the images. Then, by scrolling through the stack, the plane that showed the microtubules in the highest clarity was selected as the slice with which to do the quantification. Occasionally, the microtubules were split between two planes. When this was the case, the number of microtubules was counted in one plane, and then in the second plane, with much care taken (by consistently scrolling between the two planes) to not count any microtubule twice. Microtubules were counted using the Cell Counter feature of the ImageJ software. Any line that began at the base of the cell and extended the entirety of the cell or nearly the entirety of the cell was counted as a microtubule. Each edge of the cell (in the 2D image) was counted as having a microtubule.

QUANTIFICATION AND STATISTICAL ANALYSIS

Statistical analyses were performed using GraphPad Prism. Plots of flagellar length in Figures 2A and 2B are displayed as the mean value with error bars representing 95% confidence interval, n = 50 cells with 1 flagellum measured per cell. Figure 3B, all data points are plotted and error bars represent 95% confidence interval, n = 50 cells with 1 flagellum measured per cell. Figure 5E is plotted with all data points and error bars representing mean with standard deviation, n = 18. Figure 5F is plotted showing the mean with standard deviation, n = 100. Statistics were defined using one ANOVA for Figure 2A, Sidak's multiple comparisons test Figure 2B, Figures 5E and 5F unpaired t test, and Figure 6R Dunnett's multiple comparison.

Cell Reports, Volume *27*

Supplemental Information

Partially Redundant Actin Genes in *Chlamydomonas* Control Transition Zone Organization and Flagellum-Directed Traffic

Brittany Jack, David M. Mueller, Ann C. Fee, Ashley L. Tetlow, and Prachee Avasthi

Supplemental Material

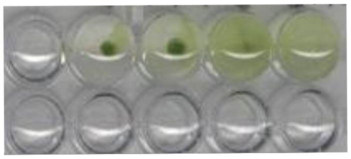

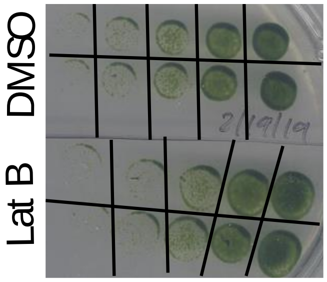

C

Cell/Volume Quantification		
Treatment	wild-type	*nap1*
1% DMSO	3.04 x 106 cell/mL	3.48 x 10^6 cell/mL
10 μM LatB	31.8 x 106 cell/mL	2.50 x 10^6 cell/mL

D

% Flagellation		
Treatment	wild-type	*nap1*
1% DMSO	72 %	73 %
10 μM LatB	87 %	82 %

Supplemental Figure 1. *Chlamydomonas* strains survive LatB treatment for at least up to 5 hours Related to Figure 2. A. wild-type and *nap1* strains treated with 10µM Lat B for 5 hours. LatB was washed out and an aliquot of the cells were added to 99 µL of liquid TAP media immediately following experiment. 5 days after re-suspension in TAP media growth was assessed and we see growth of all strains indicating wild-type and *nap1* can survive LatB treatment for at least 5 hours. **B**. wild-type and *nap1* strains treated with 10µM Lat B for 5 hours. LatB was washed out and an aliquot of the cells were plated on 1.5% TAP plates. 5 days after plating on TAP plates growth was assessed and we see growth of all strains indicating wild-type and nap1 can survive LatB treatment for at least 5 hours. **C.** Cell/volume quantification 5 days after LatB washout to show survival. **D**. Percent flagellation quantification 5 days after LatB washout cells.

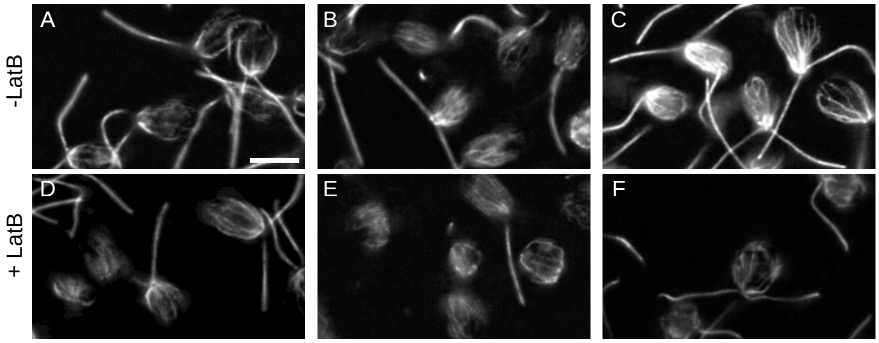

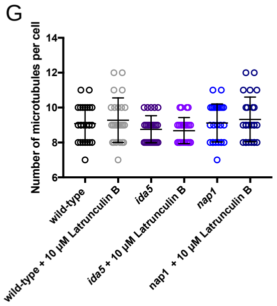

Supplemental Figure 2. Actin disruption does not cause alteration in microtubule number Related to Figure 2. A. Wild-type cells without LatB treatment. **B**. *ida5* mutants without LatB treatment. **C**. *nap1* mutants cells without LatB treatment. **D**. Wild-type cells with LatB treatment show no difference in number of microtubules. **E**. *ida5* mutants cells with LatB treatment show no difference in microtubule number.

F. *nap1* mutant cells with LatB treatment show no difference in LatB microtubule number. **G**. Quantification of number of microtubules per cell.
